# Expression of phosphorylated raf kinase inhibitor protein (pRKIP) is a predictor of lung cancer survival

**DOI:** 10.1186/1471-2407-11-259

**Published:** 2011-06-21

**Authors:** Sara Huerta-Yepez, Nam K Yoon, Angeles Hernandez-Cueto, Vei Mah, Clara M Rivera-Pazos, Devasis Chatterjee, Mario I Vega, Erin L Maresh, Steve Horvath, David Chia, Benjamin Bonavida, Lee Goodglick

**Affiliations:** 1 Unidad de Investigacion en Enferemedades Oncologicas, Hospital Infantil de Mexico, Federico Gomez, SSa, Mexico; 2 Department of Pathology and Laboratory Medicine, and Jonsson Comprehensive Cancer Center, David Geffen School of Medicine at UCLA, Los Angeles, California, USA; 3 Department of Medicine, Rhode Island Hospital; and The Warren Alpert Medical School of Brown University, Providence, Rhode Island, USA; 4 Unidad de Investigacion Medica en Inmunologia e Infectologia, CMN, La Raza, IMSS Mexico; 5 Department of Biostatistics, Department of Human Genetics, and Jonsson Comprehensive Cancer Center, David Geffen School of Medicine at UCLA, Los Angeles, California, USA; 6 Department of Microbiology, Immunology and Molecular Genetics, and Jonsson Comprehensive Cancer Center, David Geffen School of Medicine at UCLA, Los Angeles, California, USA

## Abstract

**Background:**

Raf-1 kinase inhibitor protein (RKIP) has been reported to negatively regulate signal kinases of major survival pathways. RKIP activity is modulated in part by phosphorylation on Serine 153 by protein kinase C, which leads to dissociation of RKIP from Raf-1. RKIP expression is low in many human cancers and represents an indicator of poor prognosis and/or induction of metastasis. The prognostic power has typically been based on total RKIP expression and has not considered the significance of phospho-RKIP.

**Methods:**

The present study examined the expression levels of both RKIP and phospho-RKIP in human lung cancer tissue microarray proteomics technology.

**Results:**

Total RKIP and phospho-RKIP expression levels were similar in normal and cancerous tissues. phospho-RKIP levels slightly decreased in metastatic lesions. However, the expression levels of phospho-RKIP, in contrast to total RKIP, displayed significant predictive power for outcome with normal expression of phospho-RKIP predicting a more favorable survival compared to lower levels (P = 0.0118); this was even more pronounced in more senior individuals and in those with early stage lung cancer.

**Conclusions:**

This study examines for the first time, the expression profile of RKIP and phospho-RKIP in lung cancer. Significantly, we found that phospho-RKIP was a predictive indicator of survival.

## Background

Raf-1 kinase inhibitor protein (RKIP) is a member of a conserved group of proteins called phosphatidylethanolamine-binding proteins (PEBP). RKIP was first identified by Yeung, et al., [1] and was reported to function by inhibiting the Raf-1/MEK/ERK and NF-κB proliferative and survival signaling pathways [1-3]. Based on modulation of these and other pathways, RKIP is thought to function in a number of physiological and pathological processes [4]. For example, the importance of RKIP in metastasis was demonstrated by the finding that the restoration of RKIP expression inhibits prostate cancer metastasis in a murine model [5,6] and, hence, RKIP was identified as a metastasis suppressor gene. In addition, over-expression of RKIP reverses tumor cell resistance to apoptosis by both chemotherapeutic drugs [7] and by TRAIL [8]. RKIP has also been implicated as an immune surveillance cancer gene in these studies [8]. The expression level of RKIP is down-regulated in a number of human cancers including highly metastatic prostate carcinoma [6], breast carcinoma [9], colon cancer [10] and hepatocellular carcinoma [11]. RKIP was also shown to be a prognostic marker in the pathogenesis of human prostate cancer [5], and in other cancers [10,12,13]. The mechanism of RKIP dysregulation in such malignancies is not clear. Recent findings demonstrated that Snail, a transcription factor overexpressed in many cancers and a metastasis inducer gene product (reviewed in [14,15], negatively regulates RKIP transcription and expression [16].

The inhibitory activity of RKIP on the Raf-1/MEK/ERK pathway is, at least in part, regulated by PKC-induced phosphorylation of RKIP at serine 153 [17]. The PKC family of serine/threonine kinases is a key mediator of several physiological processes including growth, differentiation, and transformation (e.g., see review [17]). Mutant RKIP that has serine 153 substituted with valine failed to associate with Raf-1 and was not phosphorylated following PKC stimulation. It has also been reported that pRKIP binds to GRK-2 and, thus, inhibits GRK-2-mediated phosphorylation of G-protein coupled receptors (GPCRs) resulting in the inhibition of receptor internalization and cell signaling integrity [18].

In the present study, we have examined the expression levels of total RKIP and pRKIP in human non-small cell lung cancers (NSCLC) on a population basis using a high-density lung tissue microarrays (TMA). Surprisingly, we found that the expression of total RKIP was similar in non-malignant bronchial epithelium, primary tumors and metastatic lesions. Moreover, RKIP neither predicted metastatic potential nor disease-specific death. In contrast, pRKIP expression was a strong predictor of outcome with relatively higher levels of pRKIP predicting a better survival compared to relatively lower expression.

## Methods

### Lung Tissue Microarray

The lung cancer tissue microarray (TMA) was constructed using archival samples from the Department of Pathology and Laboratory Medicine in the UCLA Medical Center as previously described and characterized [19]. The TMA was produced under an approved IRB protocol (protocol 02-07-011-13; UCLA Institutional Medical Review Board 2). A total of 671 patients' samples were arrayed with at least 3 spots representing each histology [19,20]. The patient demographics are shown in Table 1. In this study, we considered non-small cell lung cancer (NSCLC) of which there were 3,881 informative spots and 372 marker-informative cases.

**Table 1:** Patient demographics and Histopathologies

**Age at Diagnosis**	
Median (Range)	66 (26 - 86)
25^th ^to 75^th ^Percentile	60 - 73
**Sex**	
Male	175 (47%)
Female	197 (53%)
**Smoking History**	
Current Smoker	53 (14%)
Previously Smoked	257 (69%)
Second-Hand Smoke	11 (3%)
Non-Smoker	39 (11%)
Unknown	12 (3%)
**Histology**	
Adenocarcinoma	222 (60%)
Squamous Cell Carcinoma	106 (29%)
Adenosquamous Carcinoma	20 (5%)
Bronchioloalveolar Carcinoma	24 (6%)
**Clinical Stage**	
I	209 (56%)
II	68 (18%)
III	69 (19%)
IV	24 (6%)
Unknown	2 (1%)
**Tumor Grade**	
1	64 (17%)
2	119 (32%)
3	161 (43%)
Unknown	28 (8%)
**Tumor Size (cm)**	
Median (Range)	3.6 (0.4 - 15.0)
25^th ^to 75^th ^Percentile	2.0 - 4.7
**Lymph Node Metastases**	
Absent	224 (60%)
Present	104 (28%)
Unknown	44 (12%)
**Distant Metastases**	
Absent	274 (74%)
Present	24 (6%)
Unknown	74 (20%)

### Immunohistochemistry and TMA Scoring

Lung TMA blocks were sectioned immediately prior to Immunohistochemistry (IHC). Rabbit-anti-human phospho-RKIP (pRKIP) and rabbit anti-human total RKIP, were obtained from Santa Cruz Biotechnology, Inc. (Santa Cruz, CA) and Upstate Biotechnology (Lake Placid, NY) respectively). A standard two-step IHC protocol was used as previously described [19] using a 1:250 dilution of a 0.2 mg/ml stock of primary anti-pRKIP or 1:500 dilution of a 1 μg/ml stock of RKIP and incubating for 18 hours at room temperature. Non-immune rabbit IgG was used as a negative control and showed no staining. Staining conditions were optimized on 15 full lung cancer and normal tissue samples before the TMA was stained. A similar pattern of staining was observed for the whole tissues as with the TMA cores. For peptide competition, pRKIP peptide was purchased from Santa Cruz Biotechnology. Anti-pRKIP or anti-RKIP antibody was pre-incubated for 4 hours at room temperature with 1:250, 1:500 and 1:1000 dilutions of 100 ng/ml peptide. Antibody-peptide was then added to tissue sections that had previously been shown to express pRKIP, and incubated as described above.

The TMA was scored by a pathologist (Dr. A. Hernandez-Cueto) and spot checked by an additional pathologist (Dr. Vei Mah). All were blinded to clinical information during scoring. The percentage of relevant target epithelium expressing high (score of 3) medium (score of 2) low (score of 1) or below the level of detection (score of 0) was determined for each spot as previously described [19]. To quantify immunoreactivity of each spot, we used an integrated intensity measure using the formula: [(3x) + (2y) + (1z)]/100, where x, y, and z are the percentages of cells staining at intensities 3, 2, 1 and 0, respectively as described [19,20].

### Cell Culture

The human A549, H157 and BEA52B cell lines were obtained from the American Type Culture Collection (ATCC, Manassas, VA, USA). Cells were maintained in RPMI 1640 (Life Technologies, Bethesda, MD, USA), supplemented with 10% heat-inactivated fetal bovine serum (FBS) (Life Technologies) (to ensure the absence of complement), 1% (v/v) penicillin (100 U/ml), 1% (v/v) streptomycin (100 U/ml), 1% (v/v) L-glutamine, 1% (v/v) pyruvate, and 1% nonessential amino acids (Invitrogen Life Technologies, Carlsbad, CA, USA). The cell cultures were incubated at 37°C and 5% carbon dioxide.

### Western Blot Analysis

Cells were lysed at 4°C in RIPA buffer (50 mM Tris-HCl (pH 7.4), 1% Nonidet P-40, 0.25% sodium deoxycholate, 150 mM NaCl) supplemented with one tablet of protease inhibitor cocktail, Complete Mini Roche (Indianapolis, IN). Lysates were transferred to microcentrifuge tubes and sonicated in SONICATOR™, Model W-220F (Heat-System Ultrasonic, Inc.), for 10 seconds. The samples were then centrifuged at 12,000 × g at 4°C for 5 min. Protein concentration was quantified using the Bio-Rad protein assay (Bio-Rad Laboratories, Hercules, CA). Gel loading buffer Bio-Rad (Bio-Rad Laboratories) was added to the cell lysates, at a 1:1 volume. Samples were boiled for 5 min and were separated on 12% SDS-polyacrilamide minigels and transferred to nitrocellulose membrane Hybond™ ECL™ (Amersham Pharmacia Biotech, Germany) in Trans-Blot^® ^SD semi-dry Transfer cell System (Bio-Rad) and were subjected to Western blot analysis as previously reported [21]. Levels of β-actin were used to normalize the protein expression. Relative concentrations were assessed by densitometric analysis of digitized autographic images, performed on a Macintosh computer using the public domain NIH Image J Program.

### Statistical Analysis

All statistical analyses were performed with StatView Version 5.0 (SAS Institute, Cary, NC) or with the freely available software package, R as previously described [19,20]. The non-parametric multi-group comparison of pRKIP expression across different histolopathologic categories were done using Kruskal-Wallis test. Correlative studies of dichotomized pRKIP expression against other categorical variables were done using the Fisher exact test or Pearson χ^2 ^test. The Cox proportional hazards model was used to determine the prognostic value of various variables in a univariate and multivariate setting. Survival curves were visualized using the Kaplan-Meier method and the statistical significance between the two groups was calculated using the log-rank test.

Since each case was typically represented by more than one spot, we used the pooled mean value of integrated intensity to calculate clinical outcome [19]. We used the median value of 1 to dichotomize these values as "relatively low" or "relatively high" expression. Note that "relatively high" does not mean overexpression; these levels correspond to the normal range of pRKIP expression.

## Results

**Figure 1:**
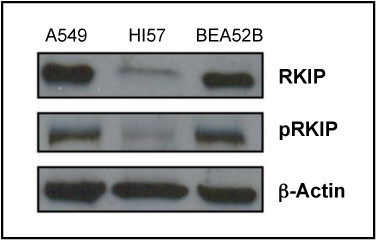
**RKIP and pRKIP protein expression lung cancer cell lines**. Different lung cancer cells lines were grown in RPMI with 10% of FBS. Total protein was extracted from the culture and then separated by SDS-PAGE and transferred onto the nitrocellulose membrane as described in Material and methods. The membrane was stained with anti-RKIP or anti p-RKIP. (lane 1) A549 cell line, (lane 2) H157 cell line, (lane 3) BEA52B cell line. The β-actin antibody was used as a loading control. The findings revealed that the differentially pRKIP expression between the different lung cell lines analyzed.

Reductions in RKIP expression has been shown to be an indicator of metastatic spread in numerous cancers [5,6,9-13,22-26] as well as a predictor of poor outcome in colon, gastric, and prostate cancer [5,10,12]. Recent evidence has also shown RKIP functionality can be modulated through phosphorylation as well [27-29]. Here, we examined the expression of RKIP and phospho-RKIP (pRKIP) in lung cancer to assess the predictive and/or prognostic power of these proteins. We first evaluated 3 lung cancer cell lines by Western blot analysis to ascertain the relative levels of RKIP and pRKIP (Figure 1). Both RKIP and pRKIP were expressed in all three cell lines, but interestingly, the level of expression was different among the cell types.

**Figure 2:**
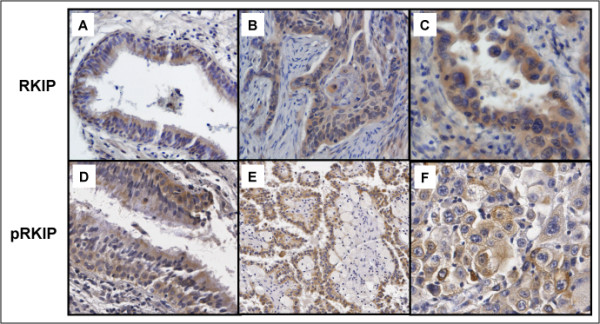
**RKIP and pRKIP protein expression in morphologically normal lung and lung cancer on tissue microarray**. Representative immunohistochemical staining for RKIP protein on (A) normal bronchial epithelium; (B) and (C) NSCLC. Representative immunohistochemical staining for pRKIP protein on (D) normal bronchial epithelium and (E) and (F) NSCLC. Magnification 100 × (A, B, D, E) and with 200 × (C, F).

To further evaluate the *in situ *expression of total RKIP and pRKIP in normal and diseased lung on a population basis, we turned to a high-density lung tissue microarray (TMA). The properties of the lung TMA have been described previously [19] and are summarized in Table 1. Of the 696 surgical specimens obtained from 671 patients, 372 primary cases of NSCLC were marker informative and linked with outcome information (disease-specific survival). The TMA consisted of a total of 5,109 spots of benign and malignant histopathologies, of which 3,881 were informative for pRKIP and RKIP. The expression of RKIP and pRKIP was localized primarily in the cytoplasm with some nuclear staining. Figure 2 show representative images of pRKIP and RKIP staining, respectively. Antibody specificity was confirmed based on negative staining with an appropriate non-immune antibody, a concentration-dependent titration of staining intensity, a lack of extracellular staining, and specific competition of antibody binding by a pRKIP peptide (see Figure S1 in Additional file 1). For this study, we focused on the cytoplasmic expression of RKIP and pRKIP. Expression was quantified using integrated measure of frequency and intensity of relevant cells of in a given spot as described in Materials and Methods.

**Figure 3:**
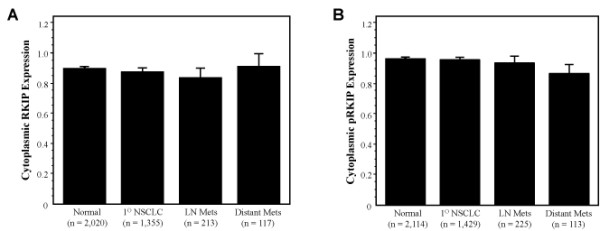
**RKIP and pRKIP expression levels as a function of tumor progression**. Columns, means, bars, SE. (A) Expression levels are represented by the integrated intensity of RKIP expression in the cytoplasm of relevant cells as described in Materials and Methods. Note, there is no statistically significant difference between any of the expression levels shown. (B) Expression levels are represented by the integrated intensity of pRKIP expression in the cytoplasm of relevant cells as described in Materials and Methods. Note, there was a statistically significant group difference of pRKIP expression (P < 0.0001) with a slight decrease in expression in metastatic cells compared to morphologically normal bronchial epithelium and primary NSCLC lesion.

We compared the levels of RKIP and pRKIP expression across histopathologies and found no statistically significant group difference among each category of NSCLC (data not shown). We next examined RKIP and pRKIP expression in normal, primary and metastatic lesions. RKIP expression remained constant for normal versus invasive cancer and metastatic lesions (Figure 3A). However, as shown in Figure 3B, when we examined pRKIP expression, we did find a slight, albeit highly statistically significant group difference (P < 0.0001) with pRKIP expression, in general, decreasing in expression from non-malignant (mean integrated intensity, 0.961) to invasive cancer (mean integrated intensity, 0.954) to lymph node (mean integrated intensity, 0.932) and distant metastases (mean integrated intensity, 0.864).

### pRKIP Expression is a Predictor of Disease Outcome

We next evaluated whether RKIP and/or pRKIP levels were predictive of clinical outcome in terms of disease-specific survival in patients with NSCLC. We used the univariate Cox model analysis and found that neither RKIP nor pRKIP expression as a continuous variable was a significant prognostic indicator of survival (P = 0.903 and P = 0.453, respectively). We then dichotomized RKIP and pRKIP expression in an unbiased fashion at the median value of 1.0 mean integrated intensity levels; values higher than 1.0 were categorized as "relatively higher expression" whereas values at or below 1.0 were categorized as "relatively lower expression". We repeated the univariate analysis using the dichotomized values of expression and found that pRKIP was a significant predictor of survival (P = 0.0125; hazard ratio = 1.53; 95% confidence interval = 1.10 - 2.15). Furthermore, using a multivariate Cox model analysis, we found that pRKIP expression remained a significant predictor of survival after correcting for the effects of stage and grade (P = 0.032, see Table 2). The individual predictive values of known clinico-pathologic variables as determined by univariate Cox model analysis are listed in Table 3. Surprisingly, and in sharp contrast, total RKIP expression levels showed no predictive value at any point of dichotomization nor in any subgroup tested (see Figure S2 in Additional file 1). Therefore, we focused on pRKIP expression for further analyses.

**Table 2:** Multivariate Cox Proportional Hazards Analysis

Variable	Hazard Ratio (95% Confidence Interval)	P Value
pRKIP Dichotomized	0.63 (0.43 - 0.93)	0.0190
Tumor Size	1.12 (1.04 - 1.19)	0.0014
Tumor Grade	1.07 (0.85 - 1.35)	0.5500
Clinical Stage	1.71 (1.45 - 2.02)	< 0.0001

**Table 3:** Univariate Cox Model Analysis of Clinico-Pathologic Variables

Variable	Hazard Ratio (95% Confidence Interval)	P Value
pRKIP Continuous	1.24 (0.454 - 1.42)	0.45
pRKIP Dichotomized	1.53 (1.10 - 2.15)	0.013
Gender	1.25 (0.921 - 1.69)	0.15
Tumor Grade	1.17 (0.947 - 1.44)	0.15
Clinical Stage	1.88 (1.63 - 2.17)	< 0.001

**Figure 4:**
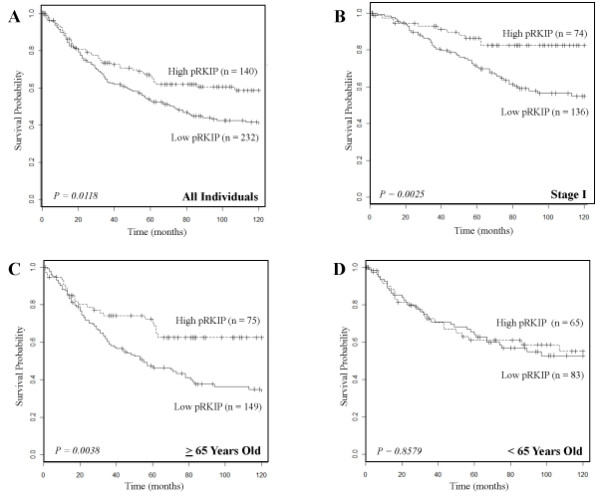
**pRKIP expression levels predict the probability of survival in individuals with NSCLC**. Shown are Kaplan-Meier survival plots for patients with NSCLC. Solid lines are higher pRKIP expression levels (mean integrated intensity > 1) and dashed lines are lower pRKIP expression levels (mean integrated intensity ≤1). n is the number of individuals in each category. (A) For all individuals represented on the TMA with NSCLC, a lower pRKIP expression level predicted a greater probability of survival compared to those with higher pRKIP levels (P = 0.0118). (B) For individuals represented on the TMA with NSCLC stage I, a lower pRKIP expression level predicted a greater probability of survival compared to those with higher pRKIP levels (P = 0.0025). (C) For individuals represented on the TMA with NSCLC who were 65 years of age or older, a lower pRKIP expression level predicted a greater probability of survival compared to those with higher pRKIP levels (P = 0.0038). (D) pRKIP expression levels yielded no predictive power for individuals with NSCLC who were younger than 65 years of age (P = 0.857).

Overall, patients with relatively higher levels of pRKIP had a distinct survival advantage compared with those with relatively lower pRKIP expression (Figure 4A, log-rank P = 0.0118). Yet in the populations dichotomized based on pRKIP expression levels, total RKIP remained constant (Figure S3 in Additional file 1). We investigated whether pRKIP expression was correlated with any known clinico-pathologic variables, and found that patients in the higher pRKIP category tended to be older than 65 years of age (P = 0.0491; data not shown) and had a higher frequency of lymph node metastases (P = 0.0484; data not shown). This was consistent with the observation that pRKIP was strongly predictive in patients older than 65 (P = 0.0033, Figure 4C), but not predictive of survival in patients younger than 65 (P = 0.8579, Figure 4D). We further assessed whether pRKIP expression levels might be an early predictive indicator in NSCLC. Indeed, pRKIP was very strongly predictive of survival in patients with early stage (I) disease (P = 0.0025, Figure 4B), but was not in patients with later stage disease (stage III and IV) (P = 0.1174, data not shown).

## Discussion

Here we present evidence that the expression levels of total RKIP was not significantly different between normal epithelium, primary non-small cell lung cancer, or metastatic lesions. Moreover, total level of RKIP had no predictive value for lung cancer patient outcome. In contrast, analyses of the expression levels of pRKIP revealed, for the first time, prognostic significance in patients with NSCLC. Specifically, individuals whose tumors had relatively higher pRKIP expression survive longer as compared to patients with relatively lower levels. It is also interesting to note that the prognostic power of pRKIP is somewhat stronger in early stage cancer (stage 1) and in patients 65 years of age or over. Although we do not yet know the mechanistic basis for this subgroup bias, we do acknowledge that such stratification might be relevant for future targeted therapy and/or early predictions of lung cancer survival. Notably, this is the first time that pRKIP has been examined in any cancer and the first indication of clinical correlate. The present findings in NSCLC are distinguished from previous findings in other cancers whereby low levels of RKIP expression suggested a poor outcome or a greater likelihood of metastasis. These findings emphasize the importance of examining the expression of both the non-phosphorylated active RKIP and phosphorylated inactive RKIP in different cancers.

The physiological significance of RKIP and pRKIP is suggested by the findings that RKIP is involved in the inhibition of the Raf-1/MEK/ERK and NF-κB cell survival signaling pathways. The interplay between these pathways and RKIP expression levels has been implicated at many steps in tumor formation and/or progression. For example, several lines of evidence demonstrate that overexpression of RKIP results in inhibition of the constitutive activation of the Raf-1/MEK/ERK and NF-κB pathways [30,31]. Further, overexpression of RKIP results in the inhibition of metastasis and invasiveness in various tumor models [6,16,32]. RKIP has also been shown to regulate drug resistance in certain systems. One clear indication of this was shown by the reversal of resistance to several chemotherapeutic drugs following overexpression of RKIP [8,31]. Consistent with this, drug-sensitive tumors were rendered resistant by knockdown of RKIP [7]. RKIP was also shown to regulate immune resistance. Overexpression of RKIP sensitized TRAIL-resistant tumor cells to apoptosis by TRAIL [8].

However, the findings with regard to NSCLC presented here, contrast with the findings in other cancers. Here we show that RKIP expression remains relatively constant in non-malignant bronchial epithelium, primary NSCLC, and corresponding metastatic lesions. Moreover, RKIP expression levels predicted neither metastasis nor survival either as a continuous or dichotomized variable. In contrast to these other studies, here, we examine for the first time, the expression levels of both total RKIP and pRKIP. Our rationale for considering pRKIP was that this level of protein regulation might yield added clinical implications especially in light of our observation that total RKIP was seemingly unchanged. It is important to note that the currently available reagents to detect total RKIP do not differentiate between phosphorylated plus non-phosphorylated forms of the protein. However, the measurement of both pRKIP and total RKIP does allow an indirect estimate of *active *(non-phosphorylated) RKIP levels. This was especially relevant given that the level of total RKIP was constant in all groups of patients dichotomized by pRKIP levels.

The underlying mechanism of the differential expression of pRKIP and RKIP is not known. Accordingly, we had expected that relatively lower levels of pRKIP might correlate with a residual higher RKIP levels that would inhibit the Raf-1 and NF-kB pathways and presumably result in a better prognosis. However, the opposite was found. Likewise, we expected relatively higher levels of pRKIP to correlate with the residual lower level of active RKIP resulting in minimal inhibition of survival pathways thus resulting in poorer prognosis. However, the opposite was found. Thus, interestingly, our present data are not concordant with these expectations.

Our findings are in apparent contradiction with other proposed functional activities of RKIP. For example, Corbit *et al.*, have reported that RKIP phosphorylation at serine 153 dissociates RKIP from Raf-1, reversing its inhibitory function [17]. Moreover, Lorenz *et al*. have reported that pRKIP binds to GRK-2 thus inhibiting GRK-2-mediated phosphorylation of G-protein coupled receptors (GPCRs) [18]. The resultant inhibition of receptor internalization has been predicted to promote cell growth and survival by maintaining appropriate extracellular signaling stimulation [18]. That relatively lower levels of pRKIP apparently correlates with a more aggressive tumor outcome in our patient population raises the intriguing question of whether a relaxation of receptor internalization - predicted with lower pRKIP - might in fact promote cell growth and survival. This interesting possibility is currently being examined. Finally, RKIP has been reported by Eves *et al*. with regard to its role in the regulation of spindle checkpoints [33]. In this study, pRKIP was found associated with the mitotic centrosomes and kinetochomes in a variety of cell types, thus implicating RKIP/pRKIP in the process of mitosis [33]. RKIP modulates the mitotic spindle assembly checkpoint by controlling Aurora B kinase activity through the Raf/MEK/ERK signaling pathway. Depletion of RKIP results in the inhibition of Aurora B kinase due to the elevated MAP kinase activity. Interestingly, several studies have shown that the excessive activation of Raf-1 MAP kinase inhibits the cell cycle leading to upregulation of cyclin-dependent kinase inhibitors and resulting in cell cycle arrest or senescence [34,35]. Nevertheless, our results and those of others [36] strongly suggest that, at least in the case of human NSCLC and melanoma, the functional interplay and balance of RKIP and pRKIP and various signaling pathways, may be more complex than seen in *in vitro *systems or other malignancies. Such tissue-specific mechanisms remain to be elucidated, and certainly should be further clarified prior to considering RKIP as a therapeutic target.

## Conclusions

In summary, this study examines for the first time, the expression profile of RKIP and phospho-RKIP in lung cancer. Significantly, we found that phospho-RKIP was a predictive indicator of disease-specific death.

## Abbreviations

ABC: avidin-biotin complex; GPCR: G-protein coupled receptors; H & E: Hematoxylin and Eosin; NSCLC: non-small cell lung cancer; PEBP: phosphatidylethanolamine-binding protein; pRKIP: phosphorylated RKIP; RKIP: Raf Kinase Inhibitor Protein; TMA: tissue microarray.

## Competing interests

The authors declare that they have no competing interests.

## Authors' contributions

All authors have read and approved the final manuscript. SH-Y, contributed to the design of the study, data interpretation, and manuscript preparation. NKY, contributed to the design of the study, data analysis, and manuscript writing. AH-C contributed to the TMA scoring. VM contributed to the TMA construction and scoring, and interpretation and data analysis. CMR-P contributed to the TMA immunohistochemistry. DC contributed to the original concept and design of the study. MIV contributed to the Western Blot analyses and data interpretation. ELM contributed to the data analyses. SH contributed to the statistical analyses of data. BB contributed to the original concept and design of the study, data interpretation, and manuscript writing and preparation. LG contributed to the original concept and design of the study, data interpretation, and manuscript writing and preparation.

## Pre-publication history

The pre-publication history for this paper can be accessed here:

http://www.biomedcentral.com/1471-2407/11/259/prepub

## Supplementary Material

Additional file 1 **Additional figures and controls**. There are 3 figures included: 1) controls for IHC staining; 2) Kaplan-Meier curve showing no predictive power for RKIP expression for patients with NSCLC; and 3) bar graph showing that total RKIP expression levels remain similar in individuals with either low or high pRKIP expression. Click here for file
