# Supplementary figures and images for: Expression of phosphorylated raf kinase inhibitor protein (pRKIP) is a predictor of lung cancer survival
Source: BMC Cancer. 2011 Jun 21;11:259. doi: 10.1186/1471-2407-11-259 (PMC3134426; doi:10.1186/1471-2407-11-259)

**Additional Files**

Huerta-Yepez, et al.


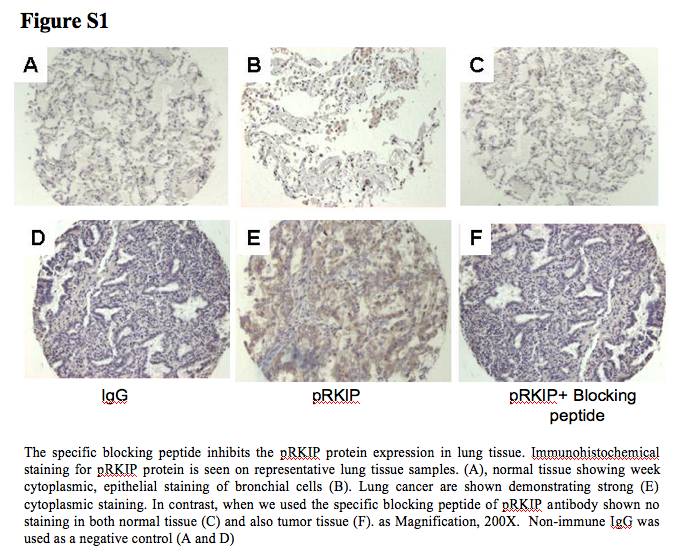


(On-line only)


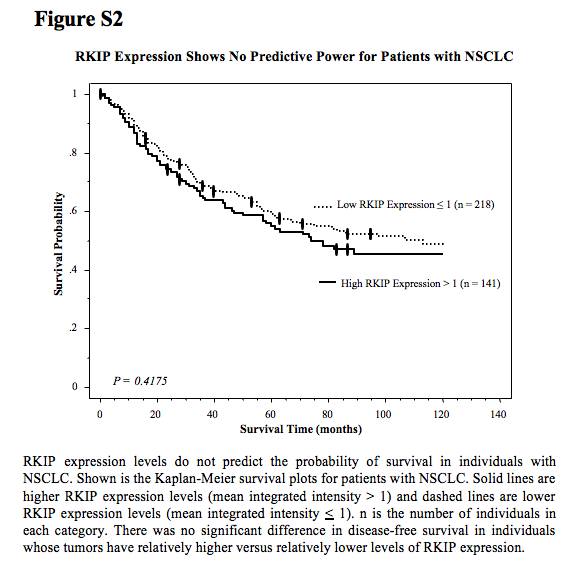


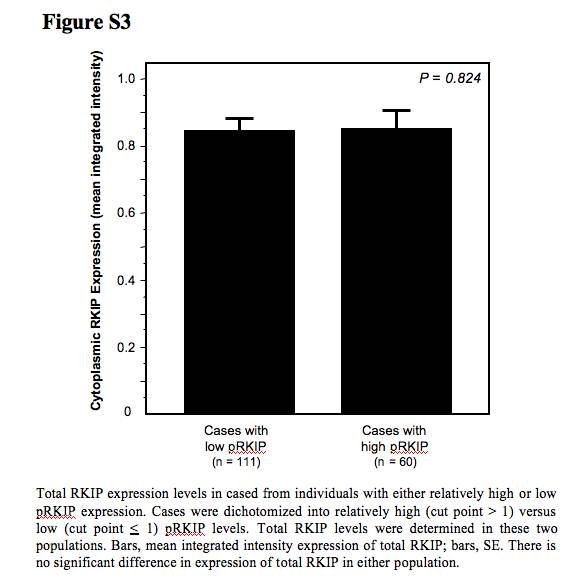

Supplement: Additional file 1 — Additional figures and controls. There are 3 figures included: 1) controls for IHC staining; 2) Kaplan-Meier curve showing no predictive power for RKIP expression for patients with NSCLC; and 3) bar graph showing that total RKIP expression levels remain similar in individuals with either low or high pRKIP expression. [file 1471-2407-11-259-S1.DOC]
